# Scanning Rate Extension of Conventional DSCs through Indirect Measurements

**DOI:** 10.3390/ma12071085

**Published:** 2019-04-02

**Authors:** Hannes Fröck, Michael Reich, Benjamin Milkereit, Olaf Kessler

**Affiliations:** 1 Chair of Materials Science, Faculty of Mechanical Engineering and Marine Technology, University of Rostock, Justus-von-Liebig-Weg 2, 18059 Rostock, Germany; hannes.froeck@uni-rostock.de (H.F.); benjamin.milkereit@uni-rostock.de (B.M.); olaf.kessler@uni-rostock.de (O.K.); 2 Competence Centre CALOR, Department Life, Light & Matter, Faculty of Interdisciplinary Research, University of Rostock, Albert Einstein-Str. 25, 18059 Rostock, Germany

**Keywords:** EN AW-6082, AlMgSi alloy, differential scanning calorimetry, fast scanning, scanning rate extension, indirect measurements, dissolution, precipitation, time-temperature-dissolution diagram

## Abstract

In this work, a method is presented which allows the determination of calorimetric information, and thus, information about the precipitation and dissolution behavior of aluminum alloys during heating rates that could not be previously measured. Differential scanning calorimetry (DSC) is an established method for in-situ recording of dissolution and precipitation reactions in various aluminum alloys. Diverse types of DSC devices are suitable for different ranges of scanning rates. A combination of the various available commercial devices enables heating and cooling rates from 10^−4^ to 5 Ks^−1^ to be covered. However, in some manufacturing steps of aluminum alloys, heating rates up to several 100 Ks^−1^ are important. Currently, conventional DSC cannot achieve these high heating rates and they are still too slow for the chip-sensor based fast scanning calorimetry. In order to fill the gap, an indirect measurement method has been developed, which allows the determination of qualitative information, regarding the precipitation state, at various points of any heat treatment. Different rapid heat treatments were carried out on samples of an alloy EN AW-6082 in a quenching dilatometer and terminated at defined temperatures. Subsequent reheating of the samples in the DSC enables analysis of the precipitation state of the heat-treated samples. This method allows for previously un-measurable heat treatments to get information about the occurring precipitation and dissolution reactions during short-term heat treatments.

## 1. Introduction

**Figure 1:**
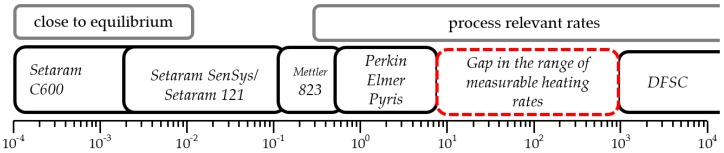
Scanning rate range of different conventional differential scanning calorimetry (DSC) devices and non-conventional fast scanning calorimeters.

Differential scanning calorimetry is an established technique used to record precipitation and dissolution reactions of various aluminum alloys in-situ, during heating [1,2] as well as cooling [3,4,5,6]. From this data, continuous time-temperature-dissolution or precipitation diagrams can be created, showing the dissolution or precipitation behaviour of the investigated alloy. Conventional DSC devices cover a large range of both heating and cooling rates, from 10^−4^ up to 5 Ks^−1^, as shown in Figure 1. In contrast, chip-based fast scanning calorimeters require a minimum heating rate of 10^3^ Ks^−1^ [7]. Thus, it turns out that, there is a gap in measurable scanning rates between about 5 and 1000 Ks^−1^. These scanning rates, though, are in an industrially relevant range. In different manufacturing steps, such as laser heat treatment [8] or welding [9], the material passes through a short-term heat treatment with heating rates up to several 100 Ks^−1^. During this short-term heat treatment, the precipitation state, and thus the mechanical properties of the material, potentially changes. Furthermore, conventional calorimeters are mainly suitable for the measurement of precipitation or dissolution reactions during linear scanning steps. However, most real heat treatments show a non-linear time temperature course. The possibilities of recording non-linear cooling processes in a DSC has been shown, but these are also limited by the device-specific maximum cooling rates [10].

It is known from the literature that information about the precipitation behaviour, during a heat treatment, can be obtained from a subsequent reheating in a calorimeter. Zohrabyan et al. [11] described an approach to obtain information from previously unmeasurable cooling rates. This approach has been used to determine the critical cooling rate of a high alloyed 7XXX aluminum alloy using the fast scanning chip calorimetry (FSC). Schumacher et al. [12] took up this approach and used the reheating to determine the enthalpy change following the cooling of samples, with extremely slow rates, down to 3 × 10^−5^ Ks^−1^. By this method, it is only possible to measure the enthalpy change during the whole cooling step. The information at which temperatures a reaction takes place, during cooling, cannot be determined. Another development of the reheating approach was done by Yang et al. [13], who carried out the reheating after multiple interrupted quenching operations, which allowed them to obtain temperature dependent data by applying the FSC technique. 

This paper aims to introduce a calorimetric method by which the dissolution and precipitation reactions, during any time-temperature curves, can be recorded in conventional DSCs. For this purpose, samples received multiple and defined previous heat treatment and subsequent reheating in a DSC. The initial heat treatments are carried out in a separate device which can realise very flexible temperature-time courses, including high heating and cooling rates, e.g., a quenching dilatometer. This produces various defined initial conditions for subsequent reheating in the DSC. The final DSC reheating curves can be used to conclude on the precipitation and dissolution reactions during the preceding heat treatments.

## 2. Materials and Methods 

### 2.1. DSC Curve Reconstruction for (Fast) Linear Heating

For the direct heating experiments in a conventional DSC, the heating rates were varied from 0.01 to 5 Ks^−1^. In order to investigate higher heating rates, a new and indirect reheating measuring methodology was developed. For this purpose, samples with the heating rate to be examined are heated to various temperatures and immediately quenched. These samples were subsequently reheated using a heating rate ideal for DSC and taking up the reheating curve. The reheating raw DSC data is treated as explained in Section 2.5. By comparing the reheating curves, which were recorded after different maximum temperatures during the previous heat treatment, conclusions about the reactions occurring during the initial heat treatment can be drawn. From the large number of investigated maximum temperatures, a virtual DSC heating curve can be reconstructed qualitatively.

To ensure the suitability of this method, some constraints must be met, as follows:

- The precipitation state of the material may not change during the cooling step. Overcritical quenching is necessary to suppress precipitation reactions during cooling.
- The precipitation state of the material may not change during the intermediate time between initial heat treatment and reheating. Store the samples in a freezer at a low temperature.

**Figure 2:**
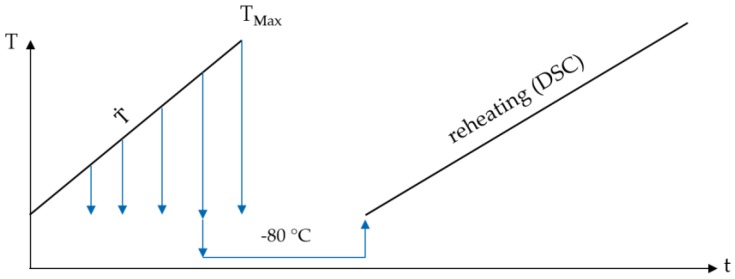
Schematic time-temperature profile of the heat treatments for the indirect measurements.

To fulfill the above requirements, samples were heated in a quenching dilatometer BÄHR 805 A/D (BÄHR Thermoanalyse GmbH, Hüllhorst, Germany), at a constant heating rate, to different maximum temperatures. After reaching the maximum temperature, without soaking, the samples were quenched using the maximum possible gas flow. The achieved cooling rates are higher than 100 Ks^−1^. Considering the actual composition of the batch of 6082, it can be assumed that 100 Ks^−1^ is above the upper critical cooling rate [6,14]. The maximum temperatures during heating were increased in discrete steps of 25 K, within a range of 150 to 575 °C. For each maximum temperature, four fresh samples in initial state T4 were used. After the initial heat treatment, the samples were placed in a freezer at −80 °C, to prevent natural ageing. These samples were finally reheated in a DSC at a heating rate of 1 Ks^−1^. The schematic time-temperature course applied for the indirect DSC measurements is shown in Figure 2.

The reheating method is used to reconstruct DSC curves. It was validated by comparing a reconstructed DSC curve with one measured by in-situ heating DSC. For this purpose, samples were heated in the dilatometer at 1 Ks^−1^ to the different maximum temperatures and quenched. The examined heating rate of 1 Ks^−1^ can be investigated by direct measurements in the DSC. This comparison can be used to establish, and justify, how a reheating curve changes when reactions take place during a previous heat treatment.

Subsequently, higher heating rates of 20 and 100 Ks^−1^ were examined by the method of indirect measurements. As a result, the heating curves of 20 and 100 Ks^−1^ were reconstructed. Those rates were not directly assessable. Table 1 shows the heat treatment parameters used for the indirect DSC measurements. In this work the results of more than 700 single DSC measurements are reported.

### 2.2. Assessment of a Non-Linear Heat Treatment by DSC on the Example of a Laser Heating and a Welding Process

In addition to the investigation of linear heating processes, the presented method is also suitable for the investigation of non-linear heat treatments. This is shown by the example of a laser short-term heat treatment, as well as for the heat affected zone (HAZ) during a welding process.

**Figure 3:**
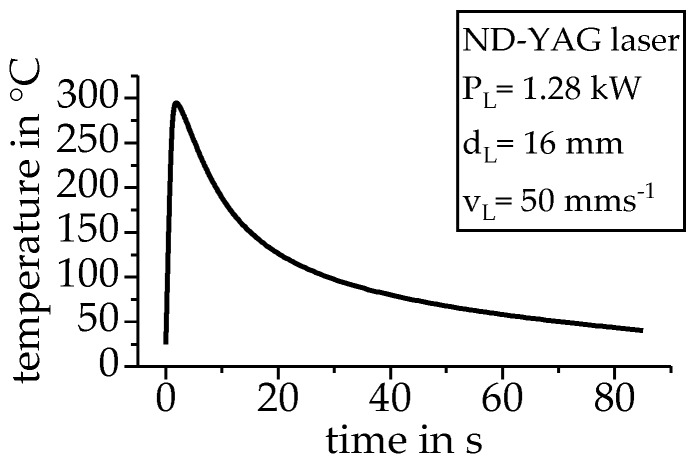
Time-temperature profile of a laser heat treatment at an aluminum extrusion profile with a wall thickness of 2 mm.

Figure 3 shows the time temperature profile recorded during a laser short-term heat treatment of an aluminium extrusion profile (EN AW-6060) with a wall thickness of 2 mm. The laser heat treatment, as well as the recording of the time-temperature-profile, was carried out at the Institute of Manufacturing Technology of the University of Erlangen-Nürnberg [15]. The laser heat treatment is characterized by a high heating rate up to several 100 Ks^−1^, with no soaking at the maximum temperature and a relatively slow, non-linear, cooling with a few 10 Ks^−1^.

Another example of a heat treatment that cannot be measured directly by calorimetry is welding. In the weld, as well as in the heat affected zone, high heating rates occur. Additionally, there is no soaking at the maximum temperature and rapid cooling follows directly. In these areas, the precipitation state, and therewith, the mechanical properties, of aluminum alloy potentially change drastically during welding. It is therefore of interest to be able to characterize the dissolution and precipitation behaviour during welding. The presented indirect method is also suitable for this problem. 

SARMAST et al. [16] show some characteristic simulated time-temperature curves in the heat-affected zone during arc welding of thick aluminum sheets. These non-linear temperature profiles were imitated again in the quenching dilatometer, interrupted at selected points by an overcritical quenching, and then frozen at −80 °C. Subsequently, the samples obtained were reheated in the DSC.

### 2.3. Investigated Aluminium Alloy

The investigated material was a hollow and quadratic aluminum extrusion profile (40 × 40 × 3) mm^3^ made from an alloy EN AW-6082. As the initial state, the naturally aged T4 state was examined. The aluminum alloy EN AW-6082 was chosen as a typical extrusion alloy, which finds application in various technical fields. The chemical composition was analysed by optical emission spectroscopy (OES) and is shown in Table 2. Samples were machined from the profile under coolant supply. Preliminary tests have shown that the temperature during machining, thereby, is kept below 30 °C, such that the naturally aged initial-state remains. The method described below is later transferred to other alloys. The compositions of the other investigated alloys are also given in Table 2. The starting material of alloy EN AW-6060 was also a hollow quadratic aluminum extrusion profile (20 × 20 × 2) mm^3^ in the natural-aged state T4, whereas, for welding, the alloy EN AW-6082 was a plate with a thickness of 10 mm in the artificial aged state T651.

### 2.4. Differential Scanning Calorimetry (DSC)

Differential scanning calorimetry has been used for two purposes, direct in-situ heating experiments for comparison with the new reheating method as well as reheating of previously heat-treated samples. Two different DSC device types were used to cover a wide range of heating rates. The slow measurements from 0.01 to 0.1 Ks^−1^ were performed in the Calvet-Type heat flow DSC Setaram S 121 (Setaram, Caluire-et-Cuire, France). For this device, typically, cylindrical samples with a diameter of 6.0 mm and a length of 21.65 mm are used. Due to the small wall thickness of the hollow profiles, such samples could not be machined. For this reason, seven samples with a diameter of 6.0 mm and a height of 3 mm were stacked into two pure aluminum crucibles. This method of stacked samples has been proven to give the same results as bulk samples [1]. The samples have a total mass of around 1580 mg. The references were also made of stacked samples of similar dimensions from high purity Al5N5 (99.9995%) aluminum.

The faster heating tests of 0.3 to 5 Ks^−1^ were performed in two power-compensated PerkinElmer DSCs, a Pyris Diamond DSC and a PerkinElmer DSC 8500 (PerkinElmer, Waltham, MA, USA). The samples for these instruments have a cylindrical shape with a diameter of 6.4 mm and a height of 1 mm. This results in a sample mass of about 80 mg. In order to keep the radiation properties of the sample as constant as possible over the whole measurement, the samples were packed in a pure aluminum crucible and covered with a pure aluminum lid. A detailed description of the different types of calorimeters used can be found in Reference [17]. All reheating experiments, for the indirect DSC measurements, were performed in the power-compensated DSC at a constant heating rate of 1 Ks^−1^.

It should be mentioned that, for a proper evaluation, the used DSC devices must be precisely calibrated regarding heat flow [18] and temperature [19]. The latter is especially true for the direct heating measurements with device-specific high heating rates, e.g., 3 and 5 Ks^−1^ in the power compensated PerkinElmer DSC, as thermal lag correction is required here.

### 2.5. Data Processing of Raw Measured Heat Flow Curves

Despite all care to maintain the symmetry between sample and the reference during the measurement, the results show a device-specific basic curvature. This base curvature can be removed by subtracting the heat flow of a baseline measurement (reference sample versus reference sample, ${\dot{Q}}_{Bl}$) from the sample measurement of heat flow (alloyed sample versus reference sample, ${\dot{Q}}_{S}$). The basic curvature of the different DSC-devices changes slightly with time, especially in the power-compensated DSCs. In order to have a close in time baseline for each sample measurement, the measurement scheme sample-baseline-sample was used.

In order to be able to compare the results of different masses and scan rates, it is necessary to normalize the recorded curves. For this purpose, the differential heat flow is normalised by the sample mass ($m_{S}$) and the scan rate (*β*) according to Equation (1) [17]. Further evaluations are carried out on the resulting excess specific heat capacity curves ($C_{p_{excess}}$).

(1) $$C_{p_{excess}}=\frac{{\dot{Q}}_{S}-{\dot{Q}}_{Bl}}{m_{S}\cdot \beta }\;\mathrm{in}\;(J\;g^{-1}K^{-1})$$

**Figure 4:**
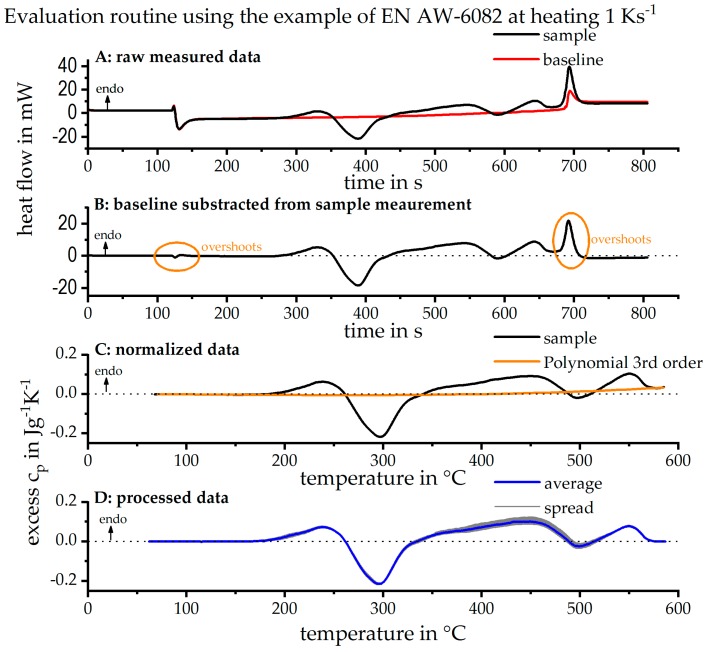
Raw data treatment of DSC heating experiments. (**A**) Raw heat flow measurement data; (**B**) subtraction of baseline measurement from the sample measurement; (**C**) normalization of the data; (**D**) processed data ready for discussion.

The raw data treatment is illustrated in Figure 4. The basis is the high-quality raw DSC data, as shown in Figure 4A. The baseline is subtracted from the raw heat flow data of the sample measurement, eliminating the device specific curvature, as seen in Figure 4B. When switching between isothermal soaking to heating and again to isothermal soaking at high temperatures, overshoot artefacts appear in the curves. These overshoot artefacts must be removed from the measurement data to avoid misinterpretations. In the next step, the data is normalised, according to Equation (1), to the unit of specific excess heat capacity. Despite due diligence when conducting the measurements, there may be small deviations between the individual measurements. This is, for example, caused by slight variations of the sample or furnace lid positions. These deviations cannot be avoided and lead to a further slight curvature of the measured data, particularly its zero level [1]. By fitting and subtracting a 3rd order polynomial, the measured data can be corrected for this zero level curvature, as shown in Figure 4C [1]. Figure 4D shows the average curve from the four processed single measurements and the scattering of the measurements. The data scattering is illustrated by plotting the DSC curves with minimum, as well as maximum, values and shading the area in-between. In the further results, only the mean value curves are shown, because the deviations between the individual measurements are small. 

It should be noted that the polynomial fit can only be performed if there is a reaction-free zone at the beginning, as well as at the end, of the graph. In order to achieve this, it is necessary to carry out the heating experiments up to the highest possible temperatures, particularly above the heating rate specific solvus temperature. This allows the completion of all the precipitation and dissolution reactions. However, melting of the sample could, potentially, damage the calorimeter. Therefore, DSC melt tests were carried out before, using ceramic crucibles. These preliminary tests show a solidus temperature of 607 °C at a heating rate of 1 Ks^−1^ for the batch 6082 T4. Thus, for the heating experiments, a maximum temperature of 600 °C has been used.

In the illustrations, the associated zero-level for each individual DSC heating curve is indicated by a dashed line. A deviation of the measurement curve above this zero-level represents the predominance of endothermic reactions, i.e. the dissolution of precipitates. A deviation below the zero-level represents the predominance of exothermic reactions, and thus the formation of precipitates.

**Figure 5:**
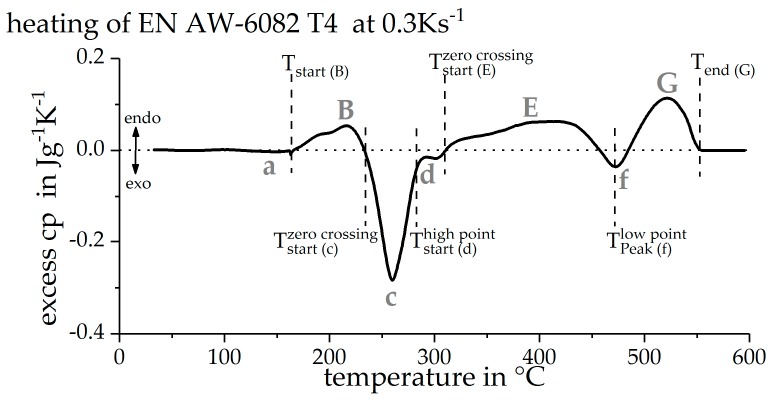
Separation and designation of the individual peaks using the example of a heating rate of 0.3 Ks^−1^.

During heating of an age hardening Al alloy in a certain metastable initial condition, typically, a sequence of alternating precipitation and dissolution reactions is seen [1,20,21,22,23]. This report intends to create continuous time-temperature dissolution diagrams. For this purpose, the single precipitation and dissolution reactions need to be separated in a certain way. This evaluation was carried out as illustrated in Figure 5 and initially described by Osten et al. [1]. For this, zero crossings of the DSC curves are evaluated. However, due to the severe superposition of the individual microstructural reactions, the applied peak separation is physically meaningless. A more meaningful separation of the distinct reactions would require kinetic modelling of the superimposed reactions, which is beyond the scope of the present paper. Therefore, at present, the applied peak separation is the method of best practice.

As a further complication, due to the severe superposition of exothermic precipitation and endothermic dissolution, some peaks do not reach the zero line. However, over the whole range of heating rates studied, it becomes clear that these reactions are occurring. In such cases, the peak temperature of the reaction is evaluated. From the characteristic temperatures determined, the associated time is calculated by means of the heating rate. These values are then plotted into the continuous time-temperature dissolution diagram for each heating rate [1].

### 2.6. Precipitation and Dissolution Reactions

In order to interpret the reheating curves, it is necessary to understand the occurring precipitation and dissolution reactions. It cannot be deduced from the DSC data which phases are precipitated or dissolved in a certain temperature range. For this purpose, further investigations, such as electron microscopy, are necessary. However, the AlMgSi alloy system has been investigated to a wide extent and the occurring peaks can be assigned to occurring reactions, based on literature data.

In the initial naturally aged state, there might be solved alloying elements in solid solution. With a slight increase in temperature, these can form clusters. The weak exothermic peak (a) is therefore generally interpreted as the precipitation of clusters resulting from the residual potential of solved alloying elements [24]. This is followed by the endothermic peak (B), which probably consists of two separate reactions. It represents the dissolution of clusters and Guinier Preston (GP)-zones that have formed during previous natural aging [25]. The dissolution reactions underlying this peak have an enormous influence on the mechanical properties. In this temperature range, the strength-increasing nano-particles of natural ageing are dissolved, causing a significant softening of the material [2]. At higher temperatures, the exothermic peaks (c) and (d) appear. These two peaks are commonly interpreted as the precipitation of the metastable phases β” and β’ [26]. Precipitates of the β” phase can provide the highest strength, while the precipitation of the phase β’ corresponds to the overaged state. The endothermic peak (E) is considered to be the dissolution of the previously formed metastable phases β” and β’ [27]. Following, the exothermic peak (f) represents the precipitation of the equilibrium phase β-Mg_2_Si [28]. At high temperatures, the endothermic peak (G) appears. This is considered to be the dissolution of the equilibrium phase β-Mg_2_Si and other remained phases [29]. With the finish of the peak (G), the DSC signal drops to zero, which indicates the scanning rate specific solvus temperature. Thus, the evaluated finish temperature of peak (G) at least has a physical meaning.

### 2.7. Previous Heat Treatment in a Quenching Dilatometer

**Figure 6:**
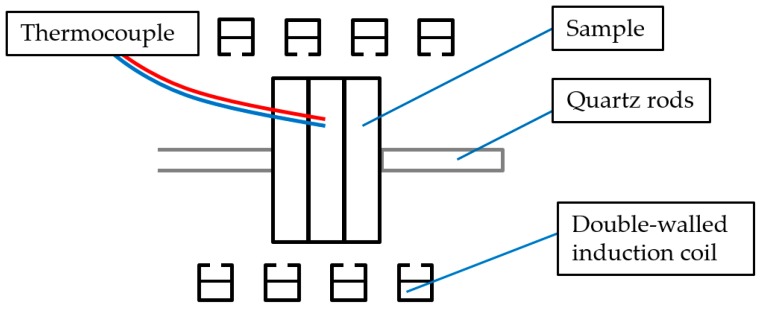
Schematic of the Bähr 805A dilatometer.

In order to realize very flexible initial heat treatments, a highly dynamic measuring setup is necessary. Conventional DSCs cannot meet these requirements. For this reason, the initial heat treatment, before reheating, is carried out in a Bähr 805A quenching dilatometer. In this device, the samples are placed in the centre of an induction coil. Defined heating rates of several 100 Ks^−1^ can be achieved. The sample temperature is continuously controlled via a spot-welded thermocouple on the surface of the sample. The samples are clamped between two quartz glass rods and held in position. The induction coil is double-walled. Gas can be passed through the inner perforated wall. Thus, cooling can take place via gas quenching and cooling rates of a few 100 Ks^−1^ can be achieved. The schematic heat treatment setup in the dilatometer is shown in Figure 6.

During heat treatment in the dilatometer, three individual DSC samples are stacked, with the thermocouple being connected to the centre sample. During the resistance spot-welding process, the sample already undergoes a certain heat treatment, with locally high temperatures changing its initial state. Thus, further investigation of this sample is not reasonable. For this reason, the centre sample, equipped with a thermocouple, is used as a temperature control dummy and is not considered for further evaluation.

**Figure 7:**
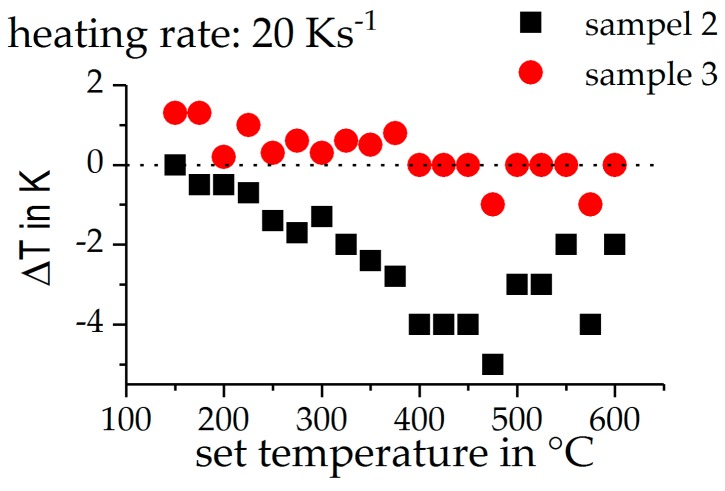
Deviation of the maximum temperature of the unregulated samples from the target temperature.

In order to evaluate the temperature distribution between the three individual samples, all three samples were provided with thermocouples in preliminary tests. Figure 7 shows the maximum deviation from the target temperature (ΔT), during a heating experiment, towards distinct maximum temperatures, using a heating rate of 20 Ks^−1^. It can be seen that the maximum temperature deviates from the target temperature by, at most, 5 K. However, most samples deviate by less than 2 K. This error is considered to be low.

## 3. Results and Discussion

### 3.1. Direct DSC-Measurements

**Figure 8:**
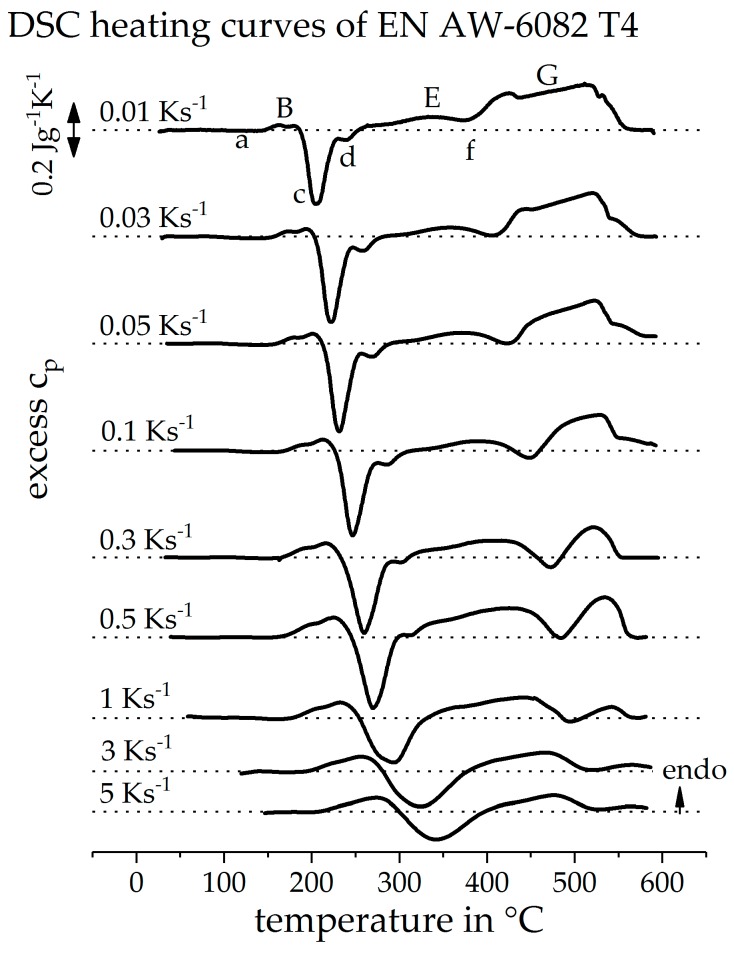
Direct DSC heating curves of EN AW-6082 T4 with the conventional DSC. Peak assignments are stated for the heating rate of 0.01 Ks^−1^.

Figure 8 shows the directly measured DSC heating curves of the alloy EN AW-6082, initially in the naturally aged T4 state. A heating rate range of 0.01 to 5 Ks^−1^ is considered. It can be seen that the peak (B) is shifted to higher temperatures with an increasing heating rate. It also appears as if the peak (B) becomes stronger with an increasing heating rate. Peak (B) represents the dissolution of clusters and GP-zones from the initial state. The content of these phases is constant before each measurement. Assuming that all these phases are dissolved during heating, this dissolution peak should also be constant in intensity over all the heating rates. Though, it is to be expected that any diffusion-controlled dissolution or precipitation reaction is suppressed kinetically. The behaviour of peak (B) is therefore explained by the overlap with the opposite exothermic peak (c). Peak (c) is also shifted to higher temperatures with an increasing heating rate. The intensity of peak (c) is also increasing, up to the heating rate of 0.5 Ks^−1^. At low heating rates, the peak temperatures of both reactions are very close to each other. For this reason, it can be considered that peaks (B) and (c) overlap very strongly at low heating rates and partly compensate each other in the sum of their heat flows. By increasing the heating rate, the peak temperatures drift apart. As a result, the overlap of the two peaks decreases and both seem to gain in intensity. With a further increase of the heating rate above 0.5 Ks^−1^, it becomes clear that peak (c) becomes blurred with peak (d) and becomes weaker overall. This is due to the increasing suppression of precipitation reactions with increasing heating rate, associated with less time for diffusion. It also becomes clear that the precipitation reaction (c + d) is suppressed earlier than the dissolution reaction (B), as already described in the literature [1].

The endothermic peak (E) is also strongly influenced by the superposition with the exothermic peaks (c), (d), and (f). The precipitation peak (f) of the equilibrium phase Mg_2_Si, at low heating rates, remains completely in the endothermic area. At this point, the strong overlap with other peaks is evident. Peak (E) appears to have a low intensity, even at low cooling rates. During this peak, the precipitates, formed in peaks (c + d), are dissolved. Peak (E) should, therefore, have a similar intensity as peaks (c + d). At low heating rates, the intensity of peak (E) is much lower than peak (c + d). It can be considered, therefore, that the dissolution of phases β”and β’ is not yet complete when the precipitation of β already begins. The endothermic dissolution reactions, as well as the exothermic precipitation reaction, overlap. Nevertheless, peak (f) shifts to higher temperatures with an increasing heating rate and is only very weak at high heating rates. It can be seen that the precipitation reaction is increasingly suppressed at higher heating rates.

The final dissolution reaction (G) also appears to lose intensity with an increasing heating rate. During this dissolution reaction, it is mainly the precipitates that have been formed during previous heating which are dissolved. This peak should become weaker if the previous precipitation reactions are suppressed with an increasing heating rate. During slow heating, coarse precipitates are formed, which must be dissolved afterwards. Increasing the heating rate should result in finer precipitates, which dissolve faster. For this reason, final dissolution of precipitates seems to be completed even at higher heating rates.

### 3.2. Validation of Indirect Rehaeting Method Versus the Known Heating Curve of 1 Ks^−1^

**Figure 9:**
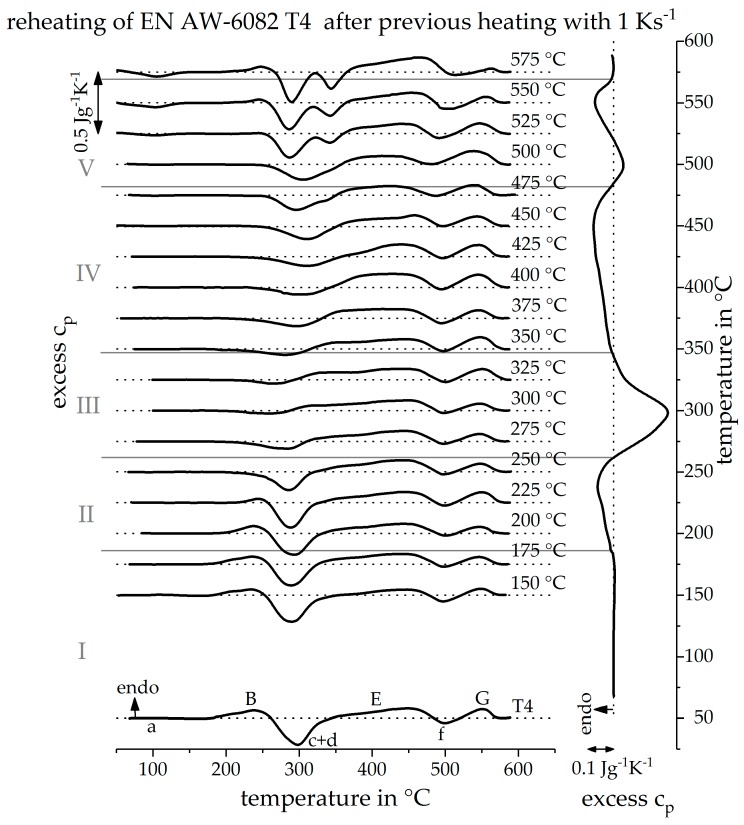
DSC reheating curves, after an initial heat treatment with a heating rate of 1 Ks^−1^, up to different maximum temperatures and the direct measured DSC heating curve at 1 Ks^−1^ in comparison.

The results of the newly developed indirect measurement methodology are presented in Figure 9 for previous heating with 1 Ks^−1^. On the left side, the reheating DSC curves after certain previous heat treatments up to different maximum temperatures, are shown. The reheating curves, after the lowest maximum temperature, during the initial heat treatment are shown at the bottom and, with increasing maximum temperature, the curves are arranged in ascending order. The maximum temperature during the initial heat treatment is given beneath each reheating curve, In order to analyze the reheating curves different sections are defined, which are separated by grey lines. The sections are labelled with Roman numerals I–V. On the right side, the directly measured DSC heating curve for 1 Ks^−1^ is shown. This curve is rotated by 90° compared to the reheating curves so that its temperature scale coincides with the maximum temperatures of the initial heat treatment and a direct comparison is possible.

In section I, up to 175 °C, no change in the DSC reheating curves can be seen compared to the initial state. It can be concluded that no reactions took place during the initial heat treatment. This is confirmed by the direct DSC heating curve.

In section II, up to 250 °C, it can be seen that the peak (B) becomes continuously weaker as the maximum temperature rises during the initial heat treatment. It can be concluded that during the initial heat treatment, some clusters and GP-zones were already dissolved. The proportion of dissolved phases rises, in section II, with increasing maximum temperature. During the subsequent reheating, less or none of these phases must be dissolved and peak (B) weakens steadily. This can also be proved by the direct measured DSC.

In the reheating curves in section III, up to 325 °C, peak (B) does not appear. All precipitates of the initial state were dissolved in the temperature range of section II. It becomes clear that peak (c + d) in the reheating curves becomes smaller in section III as the maximum temperature, during the initial heat treatment, rises. The precursor precipitates β”and β’ are formed during this peak and with a rising maximum temperature, their fraction increases. If these precipitates are already formed during the initial heat treatment, the potential for their precipitation during reheating decreases. It can be concluded that in the temperature range of section III, during the initial heat treatment, the phases β” and β’ are formed. This is again confirmed by the directly measured DSC heating curve.

In section IV, up to 475 °C, the intensity of peak (c + d) increases, again with increasing maximum temperature during the initial heat treatment. This behavior can be explained by the dissolution of the phases β” and β’. When these are dissolved during the initial heat treatment, the concentration of the aluminum solid solution, before the reheating step, increases. Thus, during reheating, the precipitates of peak (c + d) may form again. The more phases are dissolved during the initial heat treatment, the more alloying elements are solved, and the more precipitates can form during reheating. Therefore, it can be concluded, from the reheating curves, that mainly the phases β” and β’ dissolve in section IV during the initial heat treatment. This is indicated by the directly measured reheating curve.

In the reheating curves of section V, a new peak (a) occurs at about 100–150 °C, peak (B) appears weak, peak (c + d) divides into two single peaks, and peak (G) becomes progressively smaller with an increasing maximum temperature of the initial heat treatment. Due to high maximum temperatures, of at least 500 °C during the initial heat treatment in section V, a large part of the phases has already been dissolved and a high content of solved alloying elements is present in the solid solution before reheating. Already, at about 100 °C during reheating, some clusters can form, which is reflected in peak (a). It can be concluded that, during the initial heat treatment, phases with high solvus temperatures dissolve. This is supported by the directly measured heating curve in which the final dissolution peak (G) occurs.

This series of measurements has shown the feasibility of the proposed indirect DSC method. From reheating, the temperature ranges can be identified at which precipitation and dissolution reactions take place during the initial heat treatment. Therefore, by applying this procedure, unknown or unavailable DSC heating curves can be reconstructed qualitatively.

### 3.3. Reconstruction of DSC Heating Curve for 20 Ks^−1^ and 100 Ks^−1^

**Figure 10:**
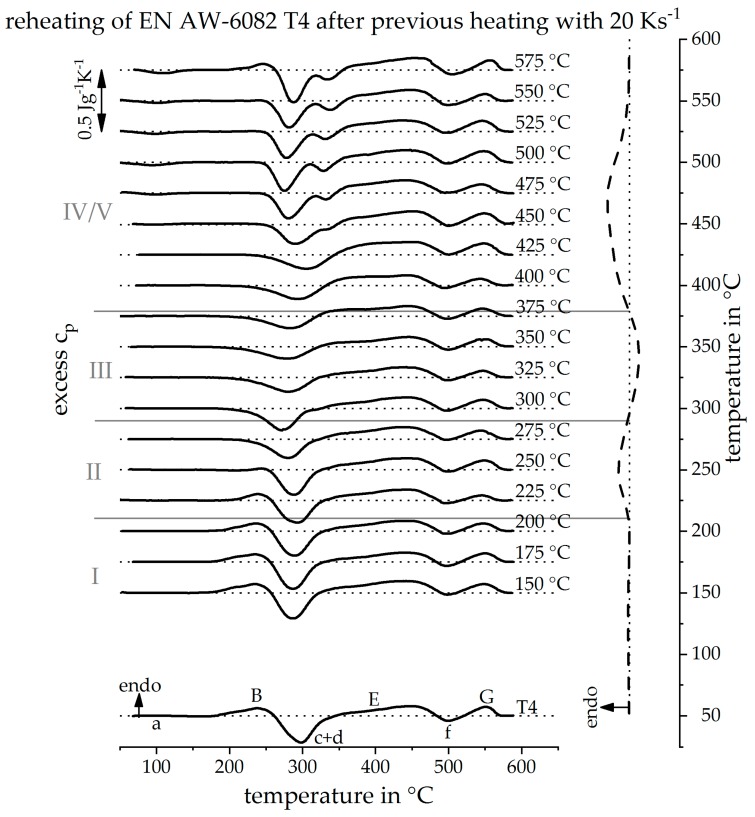
Reheating curves after a heat treatment with a heating rate of 20 Ks^−1^ up to different maximum temperatures and the resulting reconstructed heating curve at 20 Ks^−1^.

Figure 10 shows the DSC reheating curves after an initial heat treatment, with a heating rate of 20 Ks^−1^, up to different maximum temperatures (left) and the resulting, reconstructed, direct DSC heating curve at 20 Ks^−1^ (right). In section I, up to a maximum temperature of 200 °C, the reheating curves do not change in their course. It can be concluded that during heating of the alloy, with a rate of 20 Ks^−1^ up to the temperature of 200 °C, no reactions occur. This finding was adopted in the reconstructed heating curve, as seen on the right side of the diagram.

In section II, from 225 °C to 275 °C, the heating curves show that the dissolution peak (B) becomes continuously smaller with increasing maximum temperature of the initial heat treatment. It can be concluded that, during the initial heat treatment with 20 Ks^−1^, in this temperature range, the clusters and GP zones of the initial state are dissolved. For this reason, the endothermic dissolution peak (B) is plotted in the reconstructed DSC heating curve in this temperature range.

In section III, with reheating curves after an initial heat treatment with the maximum temperatures of 300 °C up to 375 °C, peak (c + d) continuously gets smaller with an increasing maximum temperature during the initial heat treatment. It can be concluded that in this temperature range, during the initial heat treatment, β” and β’ precipitates were formed. For this reason, in the temperature range of 300 °C up to 375 °C in the reconstructed DSC heating curve, an exothermic precipitation reaction of phases β” and β’ is assumed.

Sections IV and V are considered together. In the reheating curves after a maximum temperature above 375 °C during the initial heat treatment, peak (c + d), on the one hand, regains its intensity with increasing maximum temperature and, on the other hand, splits into two individual peaks (c) and (d). Furthermore, a weak precipitation reaction, peak (a), occurs during reheating at low temperatures. Peak (G) also loses some of its intensity in the reheating curves after the high maximum temperatures of the initial heat treatment. From these observations, it can be concluded that during the initial heat treatment, a dissolution of phases β” and β’ as well as phases with a higher solvus temperature has taken place. An area in which the precipitation of equilibrium phase β-Mg_2_Si dominates could not be identified. Probably, this precipitation reaction is greatly suppressed at heating with 20 Ks^−1^. Thus, from the findings, a broad dissolution reaction in the reconstructed heating curve was assumed.

**Figure 11:**
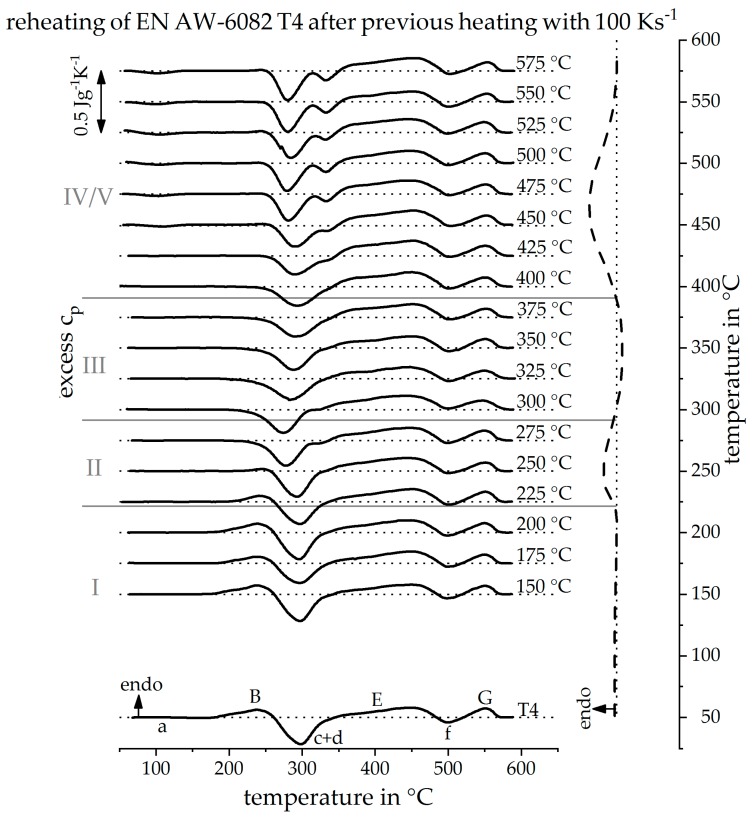
Reheating curves after a heat treatment with a heating rate of 100 Ks^−1^ up to different maximum temperatures and the resulting reconstructed heating curve at 100 Ks^−1^.

The analysis of the DSC reheating curves allowed a direct DSC heating curve to be reconstructed at the previously immeasurable heating rate of 20 Ks^−1^, as seen in Figure 10 on the right. It is important to mention, that the temperature sections for different reactions can be reconstructed quantitatively, whereas reaction intensities can only be estimated qualitatively. Therefore, the reconstructed curve is plotted as a dashed line. Due to the discrete reconstruction of the heating curve, the start or end temperatures of a reconstructed peak are determined with uncertainties. The temperature uncertainty is at least as great as the examined temperature step size. Figure 11 shows the DSC curve reconstruction for a heating rate of 100 Ks^−1^. The procedure to reconstruct the heating curve at 100 Ks^−1^ is equivalent to the reconstruction of the heating curve at 20 Ks^−1^. The resulting dissolution and precipitation sections I–V are just shifted to slightly higher temperatures.

### 3.4. The Continuous Heating Dissolution Diagram of EN AW-6082 T4

**Figure 12:**
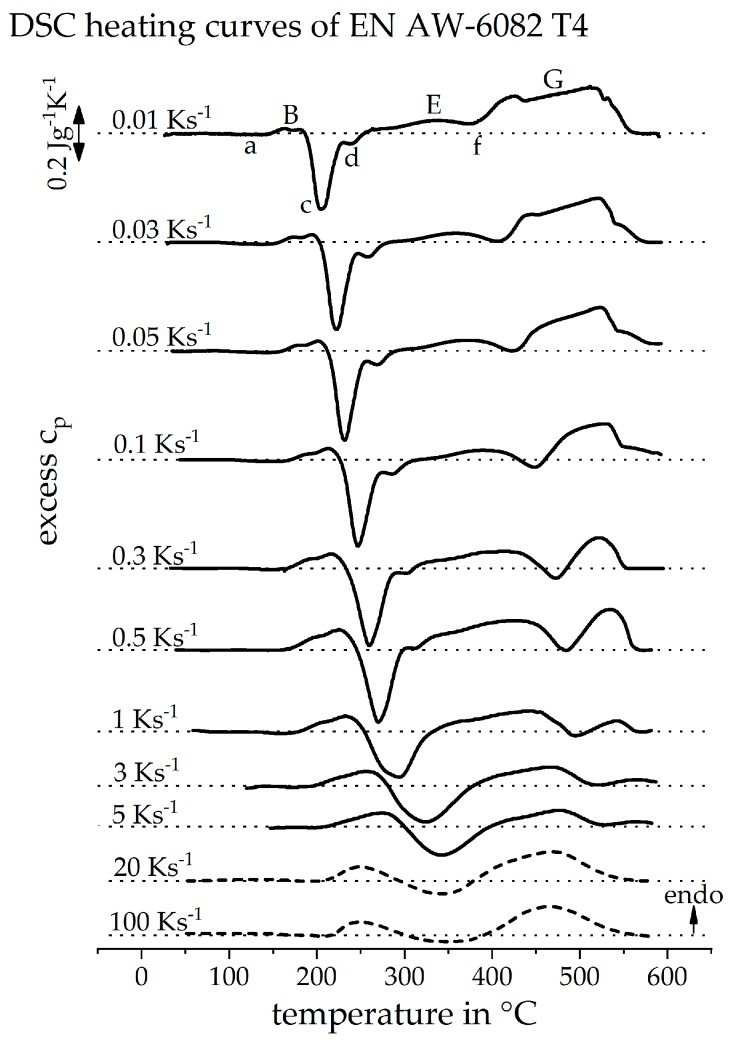
Heating curves of EN AW-6082 T4 with the conventional DSC and the reconstructed heating curves at 20 and 100 Ks^−1^.

Figure 12 shows the direct DSC heating curves of the investigated alloy EN AW-6082, in the naturally aged initial state T4, up to 5 Ks^−1^, as well as the reconstructed DSC heating curves at 20 and 100 Ks^−1^. It turns out that the reconstructed heating curves are a good fit with the directly measured heating curves. Remember that the intensities of the reconstructed DSC heating curves are only estimated qualitatively. However, it is seen that the derived temperature ranges of the individual peaks agree with those of the directly measured DSC heating curves of higher heating rates.

**Figure 13:**
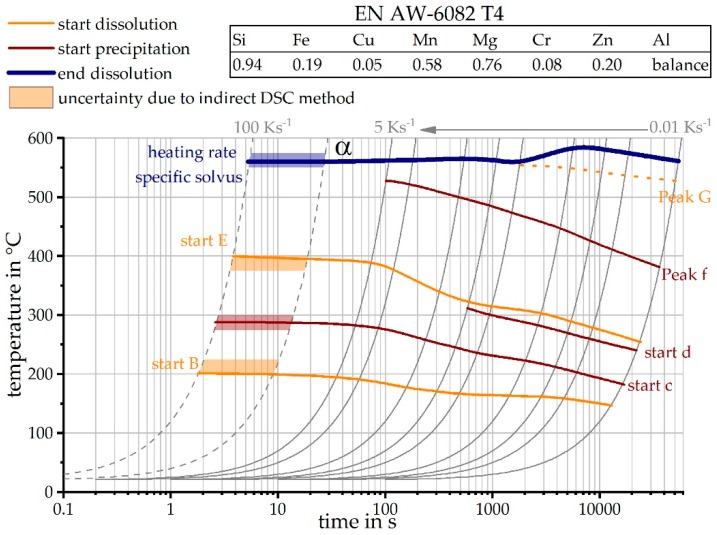
Continuous heating dissolution diagram of EN AW-6082 T4, including the findings of the indirect measurements.

Figure 13 shows the continuous heating dissolution diagram of the investigated alloy 6082 T4 obtained from the results given in Figure 12. This diagram is suitable for the selection of reasonable heat treatment parameters during production, as well as a necessary input for heat treatment simulations. The continuous heating dissolution diagrams are valid only for continuous heating steps and, therefore, are to be read at a selected heating rate (grey line) from low to high temperatures only. During heating, different reaction areas are run through. Finally, the heating rate specific solvus temperature is reached. Above this temperature for the specific heating rate, it is considered that all major alloying elements are in solid solution. Due to the proposed indirect DSC method, the heating rate range could be enlarged significantly towards higher rates.

## 4. Transfer of This Measurement Methodology

### 4.1. Transfer to Other Alloys

**Figure 14:**
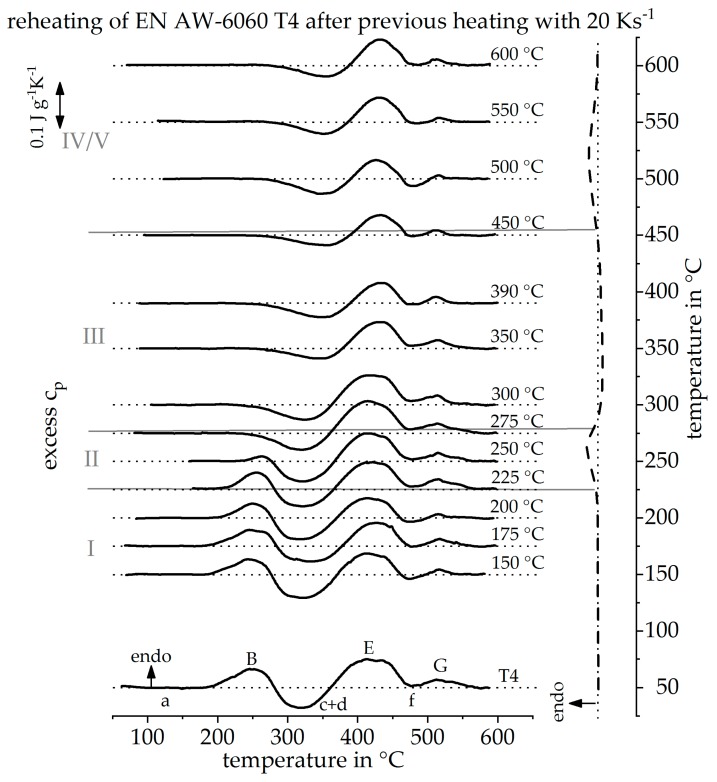
Reheating curves of the alloy EN AW-6060 T4 after a heat treatment with a heating rate of 20 Ks^−1^ up to different maximum temperatures and the resulting reconstructed DSC heating curve at 20 Ks^−1^.

The method described was transferred to other alloys as well as non-linear heat treatments. Figure 14 shows heating DSC curve reconstruction for the alloy EN AW-6060 T4 with a heating rate of 20 Ks^−1^. In comparison to the alloy EN AW-6082 T4, this alloy has a lower content of alloying elements. For this reason, the individual reactions are weaker, compared to EN AW-6082 T4. However, the main alloying elements of both alloys are the same and the initial heat treatment state is comparable. For this reason, the recorded reheating curves have a very similar course. The sequence of exothermic and endothermic peaks during heating from the initial state is identical and the individual peaks can also be assumed to refer to the same reactions. Unlike the alloy EN AW-6082, the peak (a) does not show up in this alloy during reheating, even at high peak temperatures, due to the low content of alloying elements. The procedure to reconstruct the heating curve, at 20 Ks^−1^, of this alloy is equivalent to the reconstruction of the heating curve described before.

**Figure 15:**
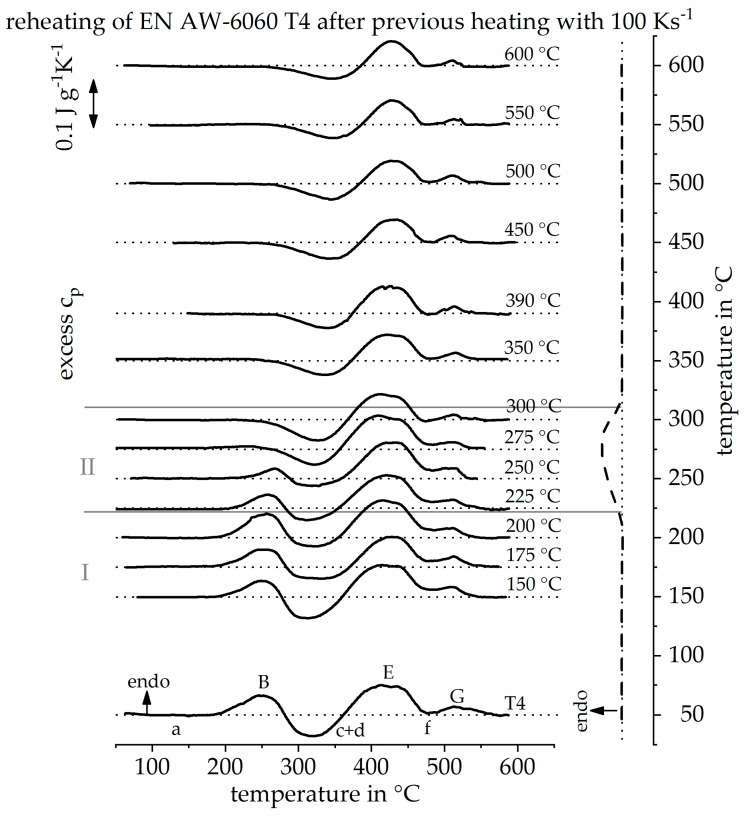
Reheating curves of the alloy EN AW-6060 T4 after a heat treatment with a heating rate of 100 Ks^−1^ up to different maximum temperatures and the resulting reconstructed DSC heating curve at 100 Ks^−1^.

**Figure 16:**
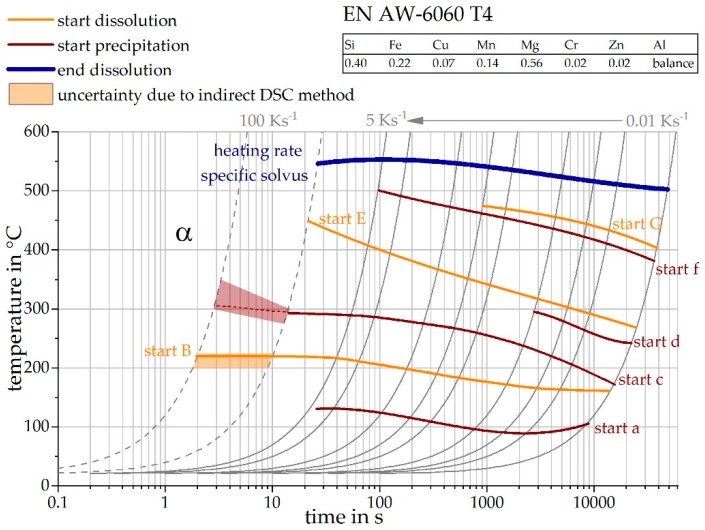
Continuous heating dissolution diagram of EN AW-6060 T4, including the findings of the indirect measurements.

Figure 15 shows the heating DSC curve reconstruction for a heating rate of 100 Ks^−1^. It is noticeable that, above a maximum temperature of 300 °C, the reheating curves show little change. From these results it can be concluded that the precipitation state no longer changes above 300 °C during this very rapid heating. It can be seen, from section II, that the T4 strength increasing precipitates are dissolved between 225 °C and 300 °C. However, the heating rate of 100 Ks^−1^ is obviously high enough to suppress further precipitation reactions. Figure 16 shows the continuous heating dissolution diagram of EN AW-6060 T4, including the findings of the indirect measurements. The heating curves of direct DSC measurements have already been published and can be seen from Fröck et al. [2]. It also becomes clear, that with this alloy, the results of the indirect measurements fit very well into the results of the direct DSC. The results have shown that the described method is also suitable for extending the bandwidth of scanning rates of other alloys.

### 4.2. Transfer to Non-Linear Heating and Cooling

The non-linear temperature profile of the laser heat treatment was analyzed using the presented indirect measurement methodology. For this purpose, DSC samples were heat treated in the quenching dilatometer, with the temperature profile given in Figure 3. At defined points, this heat treatment was interrupted by an overcritical gas quenching. Reheating curves were subsequently recorded in the DSC.

**Figure 17:**
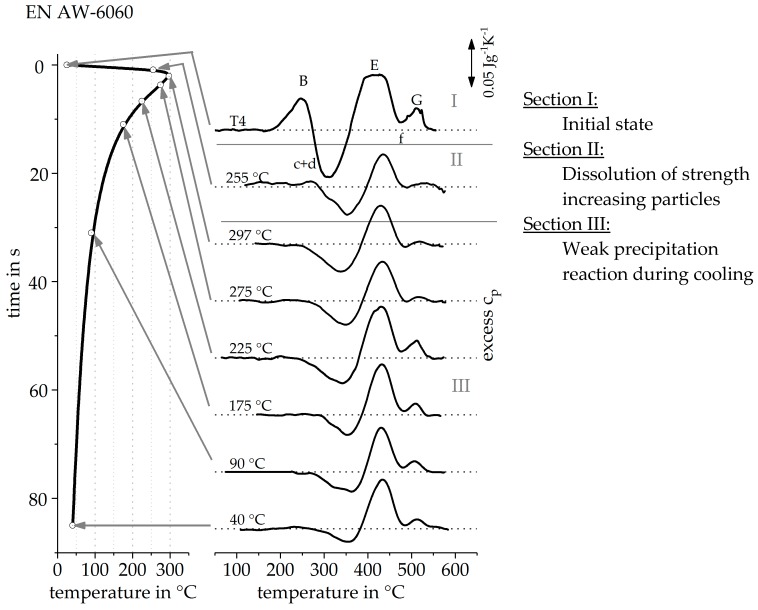
Reheating curves of the alloy EN AW-6060 T4 after an imitated laser short-term heat treatment.

Figure 17 shows the investigated time temperature profile on the left-hand, the recorded reheating curves of the alloy EN AW-6060 T4 in the middle, and the concluded precipitation and dissolution reactions on the right-hand side. The reconstruction of a DSC curve for the investigated heat treatment would be purposeless, because of the non-linearity and as well as the fact that heating and cooling are examined. The reheating curves shown in this chapter are to be read chronologically from top to bottom.

It becomes clear that, during heating of the initial state, a pronounced dissolution peak (B) takes place, as can be seen in section I. After a short-term heat treatment up to 255 °C within ≈2 s, this peak became much weaker and almost disappeared, as can be seen in section II. From this we can conclude that during the described laser heat treatment, during heating up to 255 °C, a large part of the strength increasing T4 state precipitates are dissolved. By heating to 297 °C, this peak completely disappears in the reheating curves, from which we can conclude that nearly all clusters and GP zones of the initial state have been dissolved during this heat treatment. Subsequently, the cooling starts. The reheating curves of the cooling step do not change much, as can be seen in section III. Only the peak (c + d) is slightly weaker in the reheat curves, with increasing time of the initial heat treatment. It can be concluded that a weak precipitation reaction takes place during the cooling of the laser heat treatment. Thus, alloying elements are bound in particles and the potential for precipitation during reheating decreases. 

The cooling is much slower compared to the heating, however, with respect to the upper critical cooling rate of EN AW-6060, it still has to be considered as fast for this alloy. The cooling from 297 °C to 40 °C, in total, takes 83 s, which equals an average cooling rate of 3 Ks^−1^. Compared to the results in Reference [14], the upper critical cooling rate of this batch of EN AW-6060 can be estimated to be in the order of 1 to 6 Ks^−1^. However, the reheating DSC for the cooling path indicates the occurrence of a very weak precipitation reaction.

**Figure 18:**
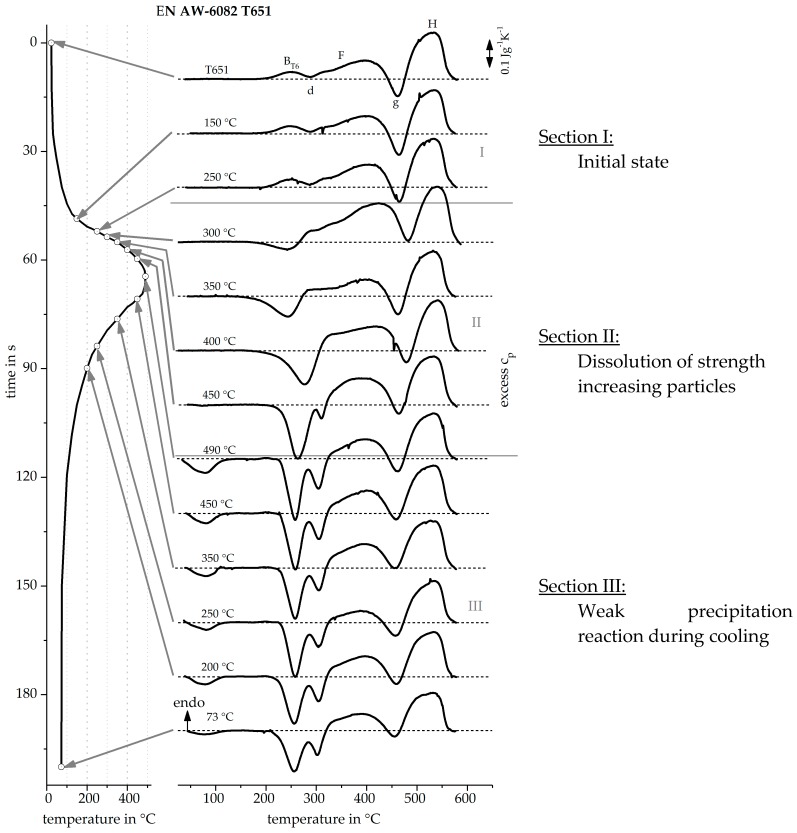
Reheating curves of the alloy EN AW-6082 T651 after heating like in the heat affected zone caused by an arc welding.

Figure 18 shows the investigated time temperature profile for the HAZ of an arc welding process on the left side [16], the recorded reheating curves of the alloy EN AW-6082 T6 in the middle, and the concluded precipitation and dissolution reactions on the right side. It is clearly visible that the reheating curves of the initial artificial aging T651 have a different course than those of the initial natural aging T4. From the reheating curves seen in Figure 18 Section I, up to a temperature of 250 °C, no obvious change is seen. From this it can be concluded that the precipitation state does not change up to this temperature. Section II, between 250 °C and 490 °C, shows that peak (B) disappears and peak (d) occurs more pronounced with increasing temperature. It can be concluded that during the HAZ heat treatment the T6 precipitates are increasingly dissolved. The dissolution of precipitates results in solved alloying elements which can form precipitates during reheating, which explains the increase of the peak (d). In Section III, the cooling, only weak reactions take place. It becomes clear that the two reactions (d) are steadily weakening. It can be concluded that a slight precipitation reaction occurs during cooling. This nicely correlates with findings on the upper critical cooling rate for this batch of EN AW-6082 of about 30 Ks^−1^ [6]. Considering the non-linear cooling within the simulated HAZ, an average cooling rate of about 12 Ks^−1^ is achieved in the relevant temperature range of 490–200 °C. As this cooling rate is below the upper critical cooling rate, a certain volume fraction of quench induced precipitation is to be expected.

## 5. Conclusions

In this work, a new indirect DSC measuring method is presented, which allows the reconstruction of DSC heating curves of fast and/or non-linear heat treatments, which were not previously assessable with DSC. Due to the combination of direct and indirect DSC, a very wide range of heating rates from 0.01 to a few 100 Ks^−1^ has been investigated, using the example of aluminum alloys, including short-term laser heat treatment and welding.

For this purpose, a large number of samples, with unknown transformational behavior, were subjected to the heat treatment of interest. This initial heat treatment must not be done in a DSC, but can be performed in any suitable controlled device, e.g., a quenching dilatometer. The heat treatment of interest was interrupted at certain points and the samples were quenched as quickly as possible in order to freeze the existing material state. These samples were then reheated at a measurable heating rate in a conventional DSC. The reconstruction of the pertinent heating curves is possible at discrete temperature steps. In this work, a temperature step of 25 K was used, which resulted in about 100 individual DSC measurements per reconstructed DSC curve.

Advantages of indirect DSC are as follows:

- Reconstruction of DSC curves for fast or non-linear heat treatments, which previously were not assessable, is now possible.
- This method is applicable for linear and non-linear heat treatments, as well as for heating and cooling.
- Temperature ranges of the main reactions can be reconstructed quantitatively.

Disadvantages of indirect DSC are as follows:

- Very time-consuming method due to many individual measurements.
- Additional device is necessary for a defined heat treatment, including the necessity of overcritical quenching to allow for process interruption.
- Validation based on a known DSC heating curve is required. This will be necessary for new alloys and new heat treatment processes. As long as alloys and processes are similar, as in in the above examples, one typical validation will be sufficient.

## Figures and Tables

**Table 1:** Heat treatment parameters for indirect measurements.

Previous Heating in Quenching Dilatometer				Reheating in DSC	
Heating-Rate	Investigated Temperature Range	Temperature Step Size	Quenching Rate	Reheating-Rate	Max. Reheating Temperature
1 Ks^−1^	150–575 °C	25 K	>100 Ks^−1^	1 Ks^−1^	600 °C
20 Ks^−1^					
100 Ks^−1^					

**Table 2:** Mass fraction of the alloying elements in the investigated alloys in %.

Alloy	Mass Fraction in %							
	Si	Fe	Cu	Mn	Mg	Cr	Zn	Al
OES EN AW-6082 T4	0.94	0.19	0.05	0.58	0.76	0.08	0.20	balance
OES EN AW-6082 T651	0.83	0.38	0.06	0.48	0.92	0.03	0.01	balance
DIN EN 573-3 (6082)	0.7–1.3	≤0.5	≤0.1	0.4–1.0	0.6–1.2	≤0.25	≤0.2	balance
OES EN AW-6060 T4	0.40	0.22	0.07	0.14	0.56	0.02	0.02	Balance
DIN EN 573-3 (6060)	0.5–0.9	≤0.35	≤0.3	≤0.5	0.4–0.7	≤0.3	≤0.2	balance
